# Fit accuracy assessment of removable partial denture frameworks produced by direct metal laser sintering – a clinical trial

**DOI:** 10.1007/s00784-025-06336-y

**Published:** 2025-04-18

**Authors:** Pedro Rodrigues Conceição, Margarida Franco, Nuno Alves, Jaime Portugal, Cristina Bettencourt Neves

**Affiliations:** 1 Dental Biomaterials Resarch Group (BIOMAT), Biomedical and Oral Science Research Unit (UICOB), Faculdade de Medicina Dentária, Universidade de Lisboa, Lisbon, Portugal; 2 Research Center on Health and Social Sciences (CARE), Polytechnic Institute of Portalegre (IPP), Portalegre, Portugal; 3 Centre for Rapid and Sustainable Product Development (CDRSP), Polytechnic Institute of Leiria (IPL), Marinha Grande, Portugal; 4 Advanced Production and Intelligent Systems (ARISE) Associated Laboratory, Porto, Portugal; 5 Research Institute for Medicines (iMed.ULisboa), Faculdade de Farmácia, Universidade de Lisboa, Lisbon, Portugal

**Keywords:** Removable partial dentures, Computer-Aided design, Computer-Aided manufacturing, Direct metal laser sintering, Accuracy

## Abstract

**Objectives:**

The purpose of this cross-over clinical study was to compare the number of framework repetitions, percentage of framework components adjusted, clinical acceptability, and fit accuracy of removable partial denture frameworks produced by the direct metal laser sintering technique (DMLS) and conventional technique.

**Materials and methods:**

For each dental arch (*n* = 26), two cobalt-chromium frameworks were produced through two protocols: direct metal laser sintering (experimental group) and conventional lost-wax casting technique (control group). The number of framework repetitions, the percentage of components that had to be adjusted, and the clinical acceptability were registered. The fit accuracy of functional components was assessed by a qualitative method using endodontic files to identify maladjustments and compared to a quantitative method based on silicone specimens digitized by micro-computed tomography. The normality was checked (Shapiro-Wilk test), and data were analyzed with McNemar, Wilcoxon and paired-t tests (α = 0.05).

**Results:**

No statistically significant differences were found between conventional and digital frameworks for most of the variables tested (*p* > 0.05) except the fewer laboratory repetitions (*p* = 0.046), higher percentage of components adjusted (*p* = 0.011), and better reciprocal arms fit (*p* = 0.044) in the frameworks produced by DMLS protocol. No statistically significant (*p* = 0.174) difference was found between the fit accuracy qualitative and quantitative assessment methods.

**Conclusions:**

The DMLS and conventional protocols were similar. Despite the DMLS protocol exhibiting a higher percentage of components adjusted, it presented better reciprocal arms fit accuracy with no framework repetition.

**Clinical relevance:**

Metal frameworks can be produced using DMLS eliminating casting problems.

## Introduction

Removable partial dentures (RPDs) are usually used to the oral rehabilitation of masticatory, phonetics and aesthetics functions when fixed dentures are not an option [1, 2]. The clinical success of RPDs depends on the framework components [3, 4], which should be fabricated to achieve an accurate fit to the corresponding oral structure enabling stability, support, reciprocity, and retention [1, 5–7].

For decades, the fabrication of RPD metal frameworks has been carried out using the lost-wax casting technique, which is susceptible to technical errors and material distortions [6, 8, 9]. These may lead to maladjustments between the RPD framework components and the oral hard and soft tissues and, consequently, functional problems such as denture deformation and oral pathologies like tooth wear, caries and periodontal disease [5, 10]. Thus, the assessment of fit accuracy of the RPDs after production is crucial to seek for maladjustments and ensure the clinical success of the oral rehabilitation [11–17].

There are several methods to assess the clinical fit accuracy of RPD metal frameworks, and no method is considered as a gold-standard [3, 18–20]. Quantitative data acquisition enables a more detailed and precise evaluation, as the one given by micro-computed tomography [11, 15, 17, 21, 22]. However, are time-consuming and complex compared to qualitative methods, which are the most described in the literature [3, 5, 20]. Additionally, direct data acquisition can be more reliable but more limited in the detail of the information collected compared to an indirect acquisition method [12, 13, 15–17].

Cobalt-chromium (Co-Cr) alloys are the most popular material used in RPD productions due to their corrosion resistance and mechanical properties such as the high yield strength and the ideal elastic modulus [20, 23–25]. The casting of Co-Cr alloys is a sensitive technique, during which certain inaccuracies may occur due to the alloy high melting range, such as pores and cracks, leading to the repetition of the previous production procedures [6, 10, 26].

Computer-aided design and computer-aided manufacturing (CAD-CAM) technology emerged to overcome the difficulties of conventional production, allowing a reduction of waste, a simplification of the procedures, an optimization in reproducibility, best mechanical properties, and patient satisfaction [4, 19, 27–31]. Many digital protocols to produce RPD metal frameworks were reported [20, 32–35]. However, the protocol that combines direct metal additive manufacturing with the conventional impression is the most frequently used in the literature showing better qualitative and quantitative fit accuracy outcomes [20]. The preference for additive methods is justified by the high complexity of the RPD metal framework design, lower waste, and lower production costs compared to milling techniques [20, 32, 34, 36–40]. The technology used for the direct additive manufacture of RPD metal frameworks is the powder-bed-fusion based on layer-by-layer assembly of powder materials using high energy beam [20, 34, 41]. The selective-laser-sintering (SLS) technique was the first to be developed in 1988 by Carl Deckard and Joe Beaman [42] but only enables the creation of point contacts between the particles once there is no melting. Consequently, SLS produces structures with high porosity and low mechanical properties [28, 37, 41]. Among the other types of additives CAM methods described to produce RPD metal structures, selective laser melting (SLM), developed in 1995, has been the most reported [20, 30, 32, 43, 44]. However, despite this technique allowing high mechanical properties [45, 46], the complete melting of the metal particles followed by cooling accumulates a large amount of residual thermal stress and can cause shrinkage in each layer compromising the RPD components fit accuracy [41, 47, 48].

In 2002, partial melting was modeled by Fisher and colleagues [49] and emerged direct metal laser sintering (DMLS) with various advantages [22, 45, 49]. Using lower energy, in the DMLS technique, there is a partial melting once only the molecules with lower melting points melt, while the molecules with higher melting points instead of melting they suffer a sintering process [36, 45, 49, 50]. Consequently, DMLS presents lower surface roughness, lower internal residual stress and higher dimensional stability, enabling lower finishing time and better fit accuracy compared to SLM [36, 39, 41, 47, 51, 52]. Also, DMLS present similar biocompatibility and mechanical properties to the ones presented by SLM [26, 28, 45, 48, 50, 53].

Moreover, few studies have yet to be reported that clinically assess the fit accuracy of multiple RPD framework components. Therefore, the main purpose of the present study was to evaluate the influence of the DMLS production protocol on the clinical assessment of RPD metal frameworks. Additionally, a second objective was defined to validate a direct qualitative assessment method for the clinical identification of maladjustment between the occlusal rests and their corresponding rest seats. The first null hypothesis tested was that the production protocol does not influence the number of production repetitions, the percentage of framework components adjusted until the correct clinical insertion, the clinical acceptability, and the fit accuracy of RPD metal frameworks. The second null hypothesis tested was that there is no difference in the efficacy of the methods to identify clinical maladjustment between the occlusal rests and their corresponding rest seats, validating the direct qualitative assessment method tested.

## Materials and methods

### Sample selection

The Ethics Committee for Health of the Faculty of Dental Medicine, University of Lisbon (FMDUL) approved the protocol of this clinical study.

The participants were selected from the university dental clinic patients with partially edentulous arches indicated for removable rehabilitation according to the list order of patients’ appointments and based on the following inclusion criteria: age over 18 years; at least one premolar or molar as a prosthetic abutment; absence of metal allergy; absence of active dental caries or grade II or III tooth mobility in the prosthetic abutments; absence of oral lesions. All twenty patients voluntarily signed a written informed consent agreement and twenty-six dental arches were considered. The sample size calculation was performed with an online tool using the results of the study conducted by Ye and colleagues. Considering the resulting Cohen’s effect (≈ 0.6), a type I error of 0.05, a type II error of 0.2, and a power of 0.8, each group should have a minimum of 25 elements [3, 54].

### Production of the RPD frameworks

A double-blind cross-over clinical study was carried out and, for each partially edentulous arch (*n* = 26), two types of RPD metal frameworks were produced defining the experimental (frameworks produced by DMLS) and the control (frameworks conventionally produced) groups. Except for Kennedy Class IV, all other classes of upper and lower arches were rehabilitated.

A preliminary impression was made for each dental arch with a universal tray and alginate impression material (Orthoprint, Zhermack GmbH, Italy) to achieve a study stone cast for design planning and customized tray fabrication. Peripheral sealing of the customized trays with godiva (Impression Compound, Kerr, USA) material was performed according to the functionality of the soft tissues. After teeth preparation, a definitive functional impression was made using an alginate impression material (Hydrogum 5, Zhermack GmbH, Italy) with special attention to the lingual frenulum. Subsequently the definitive impression was poured with type IV plaster (Elite Rock, Zhermack GmbH, Italy) to obtain the definitive cast.

In the experimental group, each definitive cast was digitized with a laboratory scanner (S600 Arti, Zirkonzahn GmbH, Italy). The Partial Planner software (Zirkonzahn GmbH, Italy) was used to survey the insertion axis and design the framework. The digital design was carried out accordingly to the basic concepts establish by McCracken [55]. A standard tessellation language (STL) file was created and sent to a laboratory production center (Sineldent, Spain). The digital buildup of the metal framework was carried out using DMLS technique using a Co-Cr SP2 alloy (EOS GmbH, Germany) and the EOSINT M270 (EOS GmbH, Germany) equipment. To release internal stresses and optimize mechanical properties, the metal framework was then subjected to a heat treatment for 45 min.

The metal framework of the control group was produced using the lost-wax technique. The same definitive cast was surveyed using a laboratory dental surveyor and the wax reliefs of the undercuts were achieved for the cast to be duplicated to a refractory cast (Rema Exact F, Dentaurum GmbH& Co. KG, Germany). Wax patterns were positioned on the refractory cast to reproduce the same planned design than the digitally designed framework. The formed patterns were eliminated in an oven (Infinity L30, Jelrus, USA). The Co-Cr alloy (Remanium G 380+, Dentaurum GmbH& Co. KG, Germany) was injected with a centrifugal induction casting machine (Ducatron Quattro, Ugin Dentaire, France).

Both metal frameworks produced by conventional and DMLS protocols were finished with hand drills and rubbers, followed by immersion in an electrolytic bath (Polytherm compact, Dentaurum GmbH& Co. KG, Germany) for three minutes and polished with brushes and paste. The finishing and polishing procedures of the two frameworks were carried out using the same definitive cast for laboratory testing. When a fail occurred during the RPD frameworks production the protocol was repeated and for each dental arch the number of repetitions was registered. All laboratory steps were made by the same technician and all the clinical steps, including impression and the following framework assessment, were made by the same researcher.

### Production control of the RPD frameworks

After the technician had attributed a random number (1 or 2) for the two RPD frameworks of each dental arch they were submitted, one at a time, to a clinical try-in. Using articulating paper (Hanel 40 μm, Roeko GmbH & Co. KG, Germany) or occlusal spray (Arti spray, Bausch GmbH & Co. KG, Germany) the components (major connector, saddles, minor connectors, occlusal rests, cingulum rests, reciprocal arms, and retentive arms) needed to adjust to allow the oral insertion of the framework were sought. The number of components adjusted until the correct insertion per framework was registered and the corresponding percentage of the total number of components was then calculated.

The clinical acceptability of the frameworks was judged according to Principles, Concepts and Practices in Prosthodontics (PCPP) guidelines [18, 56]. A RPD metal framework was described as clinically acceptable when: all rests were seated; all rigid elements touched in the abutment teeth; and the major connector did not impinge on the underlying soft tissues and had a space lower than 1000 μm evaluated with a periodontal Williams probe [18, 56].

### Fit accuracy assessment of the RPD frameworks

The fit accuracy of the RPD frameworks was always assessed by the same trained and previously calibrated researcher, using qualitative and quantitative methods for measuring the gap between the framework functional components and their corresponding supporting oral structure.

**Fig. 1:**
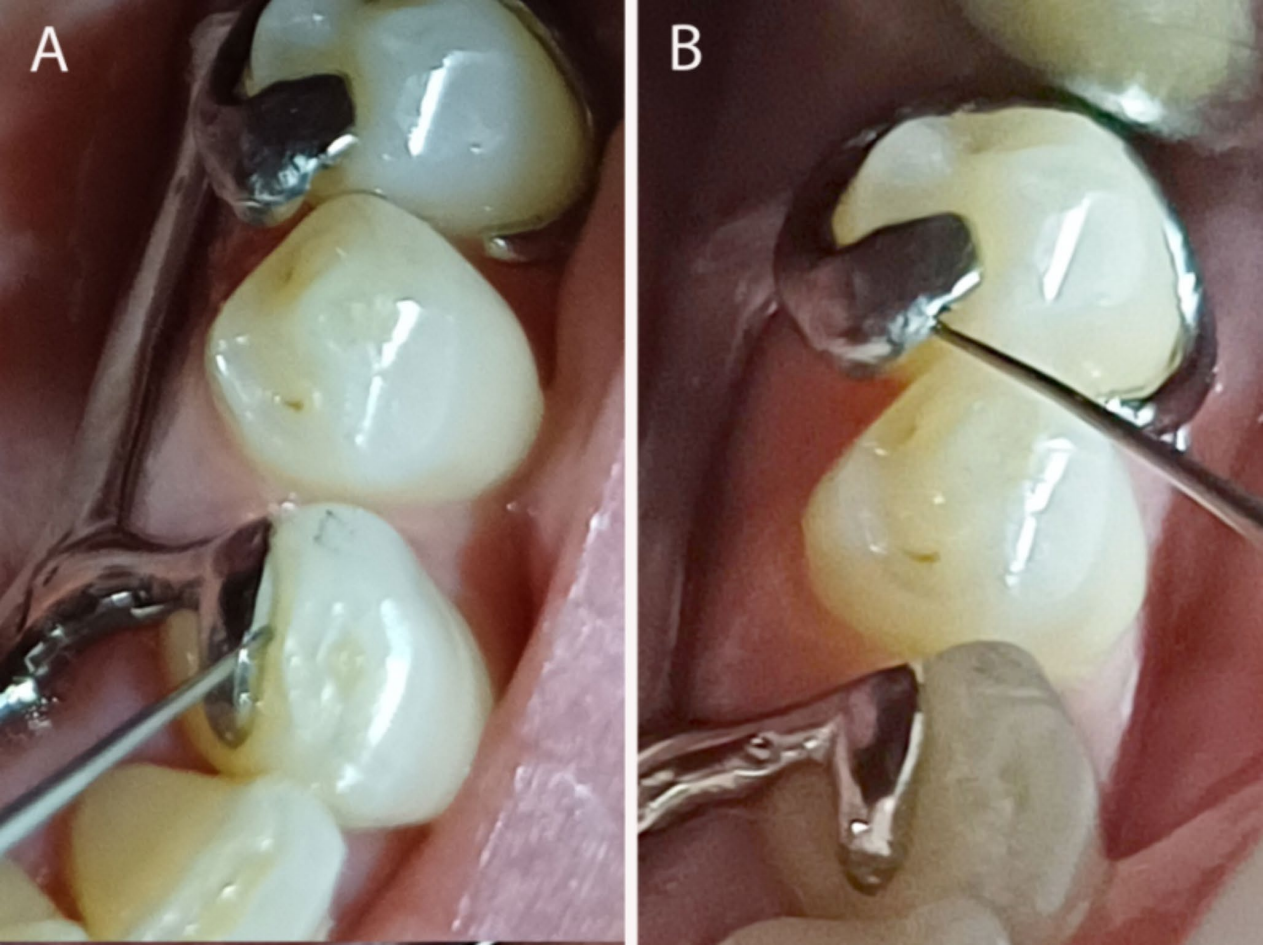
Fit accuracy assessment by direct qualitative method, inspecting the gap between teeth and framework component surfaces using an endodontic plugger size 35. **(A)** Absence of a gap thickness equal to or higher than 350 μm. **(B)** Presence of a gap thickness equal to or higher than 350 μm

A direct qualitative analog method was carried out based on the assessment of the gap between the functional components and their corresponding supporting oral structures. An endodontic Kerr file size 50 (with a 500 μm tip) was selected for the major connector evaluation and an endodontic plugger size 35 (with a 350 μm tip) was used for the other components (occlusal rests, cingulum rests and reciprocal arms) (Fig. 1). Maladjustment was considered when was detected a gap equal to or higher than the tip diameter of the instrument used.

An indirect quantitative digital assessment method was executed creating a silicone mold of the gap between the framework occlusal rests and their corresponding dental rest seats. After cleaned with a prophylaxis brush (Prophylaxis Brushlet RA 060 Soft, Edenta AG, Switzerland) the rest seats were filled with polyvinyl siloxane impression material (V-Posil Light Fast set, VOCO GmbH, Germany) and the RPD framework was positioned and maintained with finger pressure until the complete polymerization of the silicone. The obtained silicone mold of each occlusal rest was trimmed using a scalpel blade (Aesculap blade #15; Aesculap, Inc, Germany) to remove the exceeding material. The resulting specimens were individually stabilized with an orthodontic wax (Protection wax, Dentaurum GmbH & Co. KG, Germany) on the sample holder of the micro-computed tomography (micro-CT) equipment (Skyscan 1174, Bruker SA/NV, Belgium) and digitized using the following parameters: 50 KV, 800 µA, 6.6 μm image pixel size, 5500 milliseconds exposure time, 0.9 degree rotation step, and no aluminum filter. The same operator digitized all silicone specimens.

**Fig. 2:**
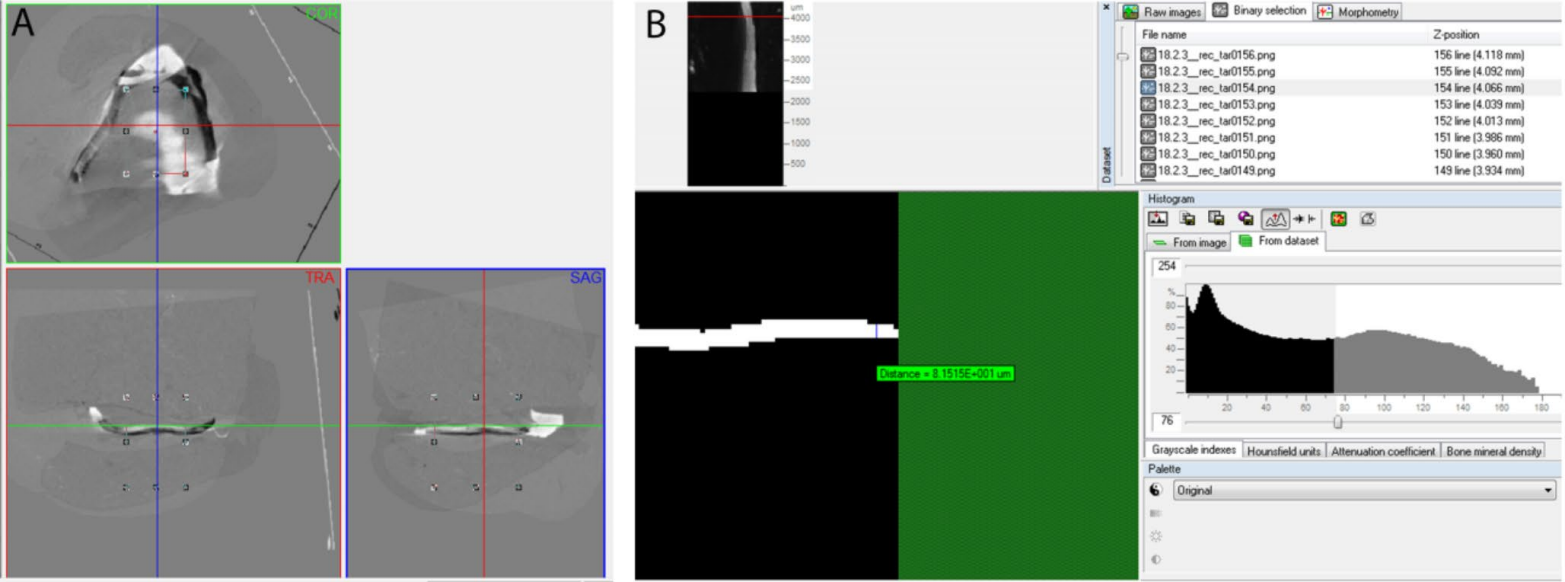
Procedures to assess silicone specimens after micro-CT digitization. **(A)** 3D Registration and analysis of two corresponding silicone molds, by overlapping of the datasets using the same VOI in the DataViewer, accordingly the three dimensional space planes (COR, coronal plane; TRA, transverse plane, SAG, sagittal plane). **(B)** Mean thickness calculation in the CT-Analyzer, after segmentation with a confirmation by a 2D measurement of the mean thickness of a silicone mold transverse segment

Specific software programs were used to reconstruct (NRecon Server Local v1.7, Bruker SA/NA, Belgium) and spatially position (DataViewer v1.5, Bruker SA/NA, Belgium) the scanned grayscale image of the silicone specimens. It was performed a 3D registration with an overlapping of the specimens using their counterpart as reference, previously defining the same volume of interest (VOI) for the reference and the target datasets (Fig. 2A). After performing the segmentation of the reconstructed image the mean thickness of the selected portion of the specimen was calculated in micrometers by a software program (CTAnalyser v1.18, Bruker SA/NA, Belgium) (Fig. 2B) and represented the thickness of the gap between the occlusal rest and the rest seat. The final thickness data per framework was obtained by the mean of all occlusal rests thickness values. The same researcher evaluated all specimens.

The quantitative data obtained for each occlusal rest by the indirect digital method was converted into dichotomous qualitative data. An absence of maladjustment was defined when the gap thickness was lower than 350 μm and a presence of maladjustment was identified when the gap was equal to or higher than 350 μm.

All data was collected by a single researcher (P.C.) after a period of training with experimental users. For qualitative data, a level of agreement greater than 82.6% (almost perfect agreement) was achieved and for quantitative data, a margin of precision error of 1.6% was obtained.

### Statistical analysis

The data was analyzed using IBM SPSS Statistics (IBM SPSS Statistics for Windows, Version 26.0, USA). Normal distribution (*p* > 0.05) was checked using the Shapiro-Wilk test. The related-samples Wilcoxon signed rank test was used to analyze quantitative non-parametric data. The paired *t*-test was applied to the quantitative parametric data. McNemar test was used for qualitative data. The level of significance was set at 95% (α = 0.05).

## Results

No patient drop-outs occurred. Considering the functional components, twenty-six major connectors, seventy-eight occlusal rests, thirty-four cingulum rests, and seventy-three reciprocal arms were evaluated. The mean number of components per framework was 17.9.

The group of frameworks produced by the conventional protocol need more repetitions (four frameworks; (*p* = 0.046) than the digital group (no repetitions). On the other hand, the DMLS protocol presented a higher (*p* = 0.011) percentage of components that needed to be adjusted until the correct insertion (median: 14.6%; interquartile range: 24.00%) compared to the conventional protocol (median: 0.0%; interquartile range: 13.50%). The number of clinically acceptable frameworks produced by the two protocols was similar (twenty-three for conventional protocol and twenty-four for DMLS protocol, *p* = 1.000).

DMLS protocol presented a lower percentage (*p* = 0.044) of reciprocal arms with maladjustment per framework (0.0% ± 0.00%) than the conventional protocol (15.7% ± 23.61%). However, no differences were found between protocols in the maladjustment of the major connector (*p* = 0.375), occlusal rests (*p* = 0.208), or cingulum rests (*p* = 0.293) (Table 1).

**Table 1:** Qualitative fit assessment results in frameworks components according to the protocols under study

		Conventional protocol	DMLS protocol	Significance (*p*)
Frameworks with major connector maladjustment		18 (69.2%)	15 (57.7%)	0.375 (McNemar test)
Occlusal rests with maladjustment per framework (*n* = 26)	Maximum	100.0%	75.0%	0.208 (Wilcoxon test)
	Minimum	0.0%	0.0%	
	Median (Interquartile range)	12.5% (37.30%)	41.5% (67.00%)	
	Mean (Standard deviation)	24.3% (± 30.54%)	35.0% (± 28.85%)	
Cingulum rests with maladjustment per framework (*n* = 17)	Maximum	100.0%	100.0%	0.293 (Wilcoxon test)
	Minimum	0.0%	0.0%	
	Median (Interquartile range)	0.0% (50.00%)	50.0% (50.00%)	
	Mean (Standard deviation)	23.5% (± 31.21%)	33.8% (± 34.17%)	
Reciprocal arms with maladjustment per framework (*n* = 26)	Maximum	66.7%	33.3%	**0.044** **(Wilcoxon test)**
	Minimum	0.0%	0.0%	
	Median (Interquartile range)	0.0% (33.30%)	0.0% (0.00%)	
	Mean (Standard deviation)	15.7% (± 23.61%)	4.5% (± 10.86%)	

**Fig. 3:**
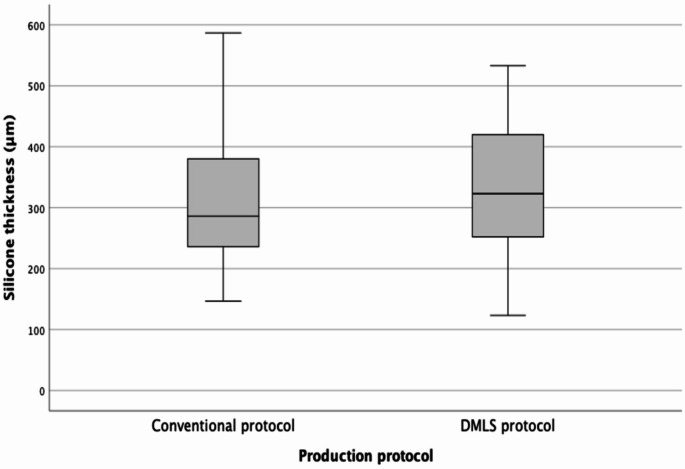
Box plots for silicone thickness values (in micrometers) in occlusal rests of the groups under study (significance using a paired t-test: *p* = 0.471). No difference (*p* = 0.174) was found between the occlusal rest maladjustments identified by the quantitative (fifty-six) and qualitative methods (forty-six) (Table 2)

No differences (*p* = 0.471) of the mean silicone thickness were found between the frameworks produced by the two protocols (DMLS protocol: 328.4 ± 106.70 μm; conventional protocol: 310.1 ± 110.89 μm) (Fig. 3).

**Table 2:** Qualitative results for occlusal maladjustment obtained by the two assessment methods under study

*n* = 148		Indirect quantitative method		Total
		NOcR without maladjustment	NOcR with maladjustment	
**Direct qualitative method**	NOcR without maladjustment	75	27	102
	NOcR with maladjustment	17	29	46
Total		92	56	148
Significance (*p*)		*p* = 0.174 (McNemar test)		

Legend: NOcR, Number of occlusal rests

## Discussion

This clinical study aimed to evaluate the clinical fit accuracy of functional components of RPD metal frameworks produced by the DMLS technique. The first null hypothesis was rejected, once despite the higher number of components needed to adjust until the correct clinical insertion, the DMLS protocol presented fewer production repetitions and fewer reciprocal arms maladjusted compared to the conventional protocol. In turn, the second null hypothesis cannot be rejected, once no difference was found between qualitative and quantitative methods for clinical identification of the framework maladjustment.

The RPDs are highly complex and custom structures that are made to increase the patients’ quality of life. Consequently, their clinical success that involves masticatory function and patient´s comfort depends on various mechanical factors, including stability, support, retention, and reciprocity, that rely on the fit accuracy of framework functional components [2, 11, 15, 20, 38]. Digital technology introduced new techniques that overcome the limitations of RPD conventional production [20, 27, 31]. This study, follow the production digital protocol most described in the literature, combining a conventional impression of the dental arch with a direct metal additive manufacturing technique for the metal framework production [20]. The use of intra-oral scanning still has limitations on RPD rehabilitation due to the demanding technique of teeth abutment and residual ridge digitization, and the impossibility of a functional impression, which is crucial for the lingual bar position [20, 57, 58]. Despite the indirect manufacturing usufructs of the CAD advantages, it does not eliminate the problems of casting and presents lower accuracy than direct manufacturing [2, 20]. Despite SLM being the most reported additive manufacturing CAD-CAM technique to produce a RPD metal framework directly, DMLS enables a better balance between the accuracy and the mechanical properties, with less energy consumption, lower internal residual stress, lower rough surface and higher dimensional stability [4, 29, 36, 39–41, 47, 51]. In the present study an experienced laboratory production center established the building parameters to optimize the DMLS production for clinical application [25, 47, 53] and, therefore, the correct chemical and mechanical properties were ensured, following the standards established by ISO standards (ISO 10271, ISO 7405, ISO 10993 and ISO 22674). Despite the temperature of the post-heat treatment was not provided as it is a commercial secret, probably was around 1100ºC [46].

Two methods were applied to assess the clinical fit accuracy of the RPD functional components: a direct qualitative method and an indirect quantitative method. Similar to Keltjens and colleagues [13] the direct qualitative method was based on a physical inspection of the gap between the structures, using a calibrated wire that allows not only a simple evaluation but also an objective identification of maladjustment (gap equal to or higher than the diameter of the instrument tip). An endodontic plugger size 35 (with a 350 μm diameter tip) was chosen due to previous findings [14] that stated an error tolerance of 311 μm, while producing a RPD metal framework, as an acceptable margin of error. The selection of the endodontic Kerr file size 50 considered the thickness of major connectors technical reliefs on the anatomical areas of the definitive cast (approximately 400 μm) [59]. For the major connector assessment, the rough surface of the Kerr files was preferred over the smoothness of the plugger files due to avoiding damage to the patient soft tissues.

In the present study, both production protocols exhibited similar clinical acceptability. However, the DMLS technique did not present laboratory failures and no production repetitions were needed, proving that CAD-CAM technology can be more consistent, predictable, and less error-prone than the lost-wax technique [27, 28, 59]. The DMLS protocol presented a higher percentage of framework components necessary to adjust than the conventional protocol (13.2% vs. 6.0%). This fact can be explained by the automatic introduction of thinner impersonalized reliefs (3 degrees) in the virtual cast after the digital surveying procedure [2]. Moreover, even though the two metal frameworks of each dental arch were previously finished by the same laboratory technician in the same definitive cast, the correction until insertion can be more accessible in the cast than in the patient’s dental arches due to the lower hardness and the wear of the definitive casts. Future investigations and calibrations on the CAD reliefs must be conducted to reduce the number of components that need to be adjusted until the correct clinical insertion.

No differences were found between the two protocols in the qualitative assessment of the fit accuracy of major connector, occlusal rests, and cingulum rests. The obtained high number of major connectors maladjusted in both production protocols (69.2% in the conventional protocol and 57.7% in the DMLS protocol) is a reminder of the ongoing challenge in the fit of RPD metal frameworks, supported by previous studies that found a mean gap discrepancy of major connectors between 310 and 640 μm [4, 15]. In the present study, the percentage of the occlusal and cingulum rests with maladjustment fluctuated between 23.5% and 35.0%, which is in line with the statements of other authors [11, 13, 15, 18], supporting that the fit of the RPD metal frameworks is a challenge. Stern and colleagues [11] concluded that 21% of the occlusal rests had no contact with the respective dental seat and Frank and colleagues [18] found that 32% of the metal frameworks showed poor fit.

The reasons for the maladjustments in the conventional protocol were already reported [10], and the discrepancies found in DMLS protocol can be attributed not only to the distortions of the thermal heat dissipation but also to the building parameters, support, and orientation that were set [40, 47, 51]. However, the DMLS protocol demonstrated lower maladjustment of the reciprocal arms (4.5%) than the conventional protocol (15.7%). This outcome is in line with the findings of the case report study conducted by Muehlemann and colleagues [4] and underscores the potential to enhance the fit accuracy of the RPD functional components, which can be attributed to the thinner reliefs during the CAD procedure, instilling hope for future improvements.

Consistency was observed among the two different methods to assess the fit accuracy of occlusal rests, once also no difference was found between the two production protocols when the quantitative method was applied. The gap discrepancies found are in the range (82 to 390) reported by previous studies for the RPD frameworks produced by direct additive manufacturing techniques [20, 35]. Focusing in the production by DMLS, our findings are supported by the results of previous in vitro studies [39, 40].

Due to their great importance in stability and support of the RPDs, the occlusal rests are the most used components to assess the fit accuracy of metal frameworks [7, 11, 15, 20, 38]. Therefore, in this study an indirect quantitative digital method already reported in the literature [17, 22] was applied to evaluate the gap between the occlusal rests and the corresponding dental rest seats with more detail. Although the direct qualitative analog method only explored the peripheral edge of the occlusal rest and not the gap under it, as the indirect method did, no statistically significance differences were found between them in the ability to identify maladjustments correspondents to gaps with a discrepancy equal to or higher than 350 μm. Consequently, the proposed direct qualitative method, similar to the one described by Keltjens and colleagues [13], can be an economical and essential technique for a simple and fast clinical assessment of the fit accuracy of the RPD frameworks occlusal rests.

During the present study, some difficulties and limitations were found due to the complexity of the applied procedures. The same researcher consistently executed the frameworks assessment after a training and calibration period, avoiding bias in the results. The sensitive selection of the VOI, after the registration, was always done to exclude areas where the silicone molds were bent due to the delicate stabilization on the orthodontic wax or presented porosity due to the impossibility of removing all the humidity and air during the application of the silicone by drying and mixing tip vibration. Only one specimen was so crumpled that it was not possible to overlap with its pair, leading to the exclusion of both. In three silicone specimens choosing a VOI without porosity was impossible, excluding the correspondent pairs. Moreover, considering the segmentation value defined, the micro-CT technique requires some attention to appreciating absolute measurements, despite being a reference in relative measurements [21]. Considering the particularities in the surface of the RPD frameworks produced by the DMLS protocol, such as the high details, minimum oxidation, and step effect, the researcher was not truly blind to all frameworks [41, 45].

Owing to the complexity and the limitations of the clinical investigations on this issue and the probability of an error type II (β = 0.8), more studies with larger samples are needed to confirm the viability of the DMLS protocol to produce RPD frameworks.

## Conclusions

Despite the limitations of the present study, it is possible to conclude that the DMLS technique is a viable alternative to produce RPD metal frameworks and the direct qualitative method using endodontic files is a viable technique for a simple and fast clinical fit assessment of RPDs occlusal rests.

## Data Availability

No datasets were generated or analysed during the current study.
